# Anesthetic Considerations in a Patient with Myotonic Dystrophy for Hip Labral Repair

**DOI:** 10.1155/2017/6408956

**Published:** 2017-02-20

**Authors:** Ramon Go, David Wang, Danielle Ludwin

**Affiliations:** Department of Anesthesiology, New York-Presbyterian, Columbia University Medical Center, New York, NY, USA

## Abstract

Myotonic Dystrophy (DM) affects multiple organ systems. Disorders such as hyperthyroidism, progressive musculoskeletal weakness, cardiac dysrhythmias, hypoventilation, and cognitive-behavioral disorders may be present in these patients. Thorough preoperative assessment and anesthetic planning are required to minimize the risk of anesthetic complications. Patients with DM can exhibit exquisite sensitivity to sedatives, neuromuscular blocking agents, and volatile anesthetics, resulting in potential postoperative complications. There is limited literature available on successful anesthetic techniques for the DM patient. We present this case report to add to our current fund of knowledge.

## 1. Introduction

Myotonic muscular dystrophy (dystrophia myotonica) (DM) is a rare musculoskeletal disease with a prevalence of 1 in 8,000 [1]. This genetic disease requires significant considerations for patients in the perioperative period. Here we discuss the case of a patient with DM for labral hip repair and review the literature on the management and anesthetic concerns of DM.

## 2. Case Report

Our patient is a 58-year-old Caucasian man weighing 92.9 kg, 175 cm tall, with a history of type 1 DM who presented for repair of a hip labral tear. His past medical history was significant for obstructive sleep apnea (OSA), gastroesophageal reflux disease, bicuspid aortic valve, bipolar disorder, obsessive-compulsive disorder, and cataracts. His only prior anesthetic exposure was for cataract surgery and he had no complications. However, the patient's daughter, who also had DM, experienced severe respiratory depression following general anesthesia, requiring intensive care unit (ICU) admission postoperatively. Primary considerations in relation to anesthesia include the disease's association with cardiomyopathy and cardiac conduction abnormalities, sensitivity to respiratory depression and ventilatory weakness, prolonged gastric emptying, and myoclonus triggered by stimuli such as hypothermia and specific medications.

Following the application of standard ASA monitors, a combined spinal and epidural anesthetic technique was performed successfully. Fifteen mg of isobaric bupivacaine was injected into the subarachnoid space at the L4-L5 interspace and an epidural catheter was inserted immediately. The spinal level was tested and found to be at a T10 dermatomal level. External pacer/defibrillator pads were applied and an arterial line was used for continuous blood pressure monitoring and to facilitate arterial blood gas measurements in the event of pulmonary compromise. A thermometer was placed in the patient's axilla for continuous monitoring. The operating room's ambient temperature was increased, a forced-air warming blanket was applied to the patient, and a fluid warmer was connected to his intravenous line. Intraoperatively, the patient received small (0.5 to 1 mg) boluses of midazolam titrated for a Richmond Agitation-Sedation Scale (RASS) of −3. For the 3 hour and 43 minute procedure, the patient received a total of 10 mg of midazolam and 50 mcg of fentanyl. Forty-five minutes into the surgical procedure the surgeon requested further relaxation of the patient's hip muscles and the epidural catheter was subsequently bolused with 5 mL of 2% lidocaine. Two hours into the procedure, another 5 mL of 2% lidocaine was bolused into the epidural. No complications were noted in the intraoperative period. The patient was transported to the postanesthesia care unit (PACU) with continuous SpO_2_, ECG, and blood pressure monitoring. A written consent was obtained from the patient for this case report.

## 3. Discussion

Two genes have been identified as playing a role in the development of DM. A CTG expansion in* DMPK* gene results in type 1 (DM1), while an expansion in the ZNF gene results in type 2 (DM2) [1, 2]. Although the functions of these genes are unknown, the CTG expansion of either gene results in faulty communication within the cell. The severity of the disease appears to correlate with the expansion repeats. DM affects multiple organ systems and patients may present with different symptomatology (see Table 1). A thorough assessment of the patient is critical to successful perioperative management (see Table 2). Poor preoperative assessment or undiagnosed DM in a surgical patient can lead to morbidity and mortality in the perioperative period.

## 4. Central Nervous System and Behavioral

DM is associated with cognitive impairment, anxiety, and bipolar disorder which may limit perioperative cooperation and preparation [1]. Patients commonly demonstrate hypersomnia or excessive daytime somnolence independent of neuromuscular respiratory compromise [3]. Together, these CNS effects increase sensitivity to sedatives, anxiolytics, and analgesics that put the patient at high risk for compromised ventilatory drive and potential for aspiration. Patient's sensitivity to short-acting opioids should be assessed prior to administering long-acting opioids.

## 5. Pulmonary

Lee and Hughes demonstrated abnormalities in lung function tests in 90% of patients. In severe cases, an obstructive pattern is seen [4]. A diminished response of respiratory muscles to respond to increasing carbon dioxide (CO_2_) levels has also been observed, suggesting that the shift in the CO_2_ response curve may perhaps not only be due to increased sensitivity to opiates [4]. Radiological evidence of abnormal swallowing has also been observed, likely explaining episodes of aspiration pneumonia [4]. The pulmonary effects of DM can be significant as shown in a clinical study of 219 patients undergoing surgical procedures by Mathieu; 89% of all complications were pulmonary in nature [1, 5]. Furthermore, patients with DM2 who have less pulmonary involvement have been shown to have less perioperative involvement as compared with DM1 [6]. Both DM1 and DM2 are associated with a high prevalence of sleep-disordered breathing. Sleep studies have documented OSA in 69% of DM1 and 43% of DM2 [3]. Careful monitoring in the postoperative period and assessing the patient's ability to protect his/her airway and aggressive pulmonary hygiene are crucial to preventing anesthetic complications in the DM patient.

## 6. Cardiovascular

The cardiovascular effects of DM have been well established. Dense granules in the mitochondria of cardiac myocytes result in necrosis, fatty infiltration, and fibrosis resulting in hyperexcitability of the cardiac conduction system [7]. Atrioventricular conduction abnormalities in patients with DM have been shown to increase the risk of ventricular arrhythmias. Benhayon et al. found 32% of DM patients with atrial fibrillation [8]. Patients with DM1 are more likely to have conduction disease and have higher all-cause mortality as compared with DM2 [8]. In a 20-year study of 171 patients with DM, sudden death was the most common cause of patient demise at 41.7%, while respiratory complications were associated with 29.2% of deaths [9]. Hypertension, presence of palpitations, right bundle branch blocks, bifascicular blocks, and a “severely abnormal” EKG were identified as risk factors for sudden cardiac death (SCD) in DM patients [9]. In a 2004 study of 382 DM1 patients by Bhakta et al., abnormal electrocardiographic findings correlated structural heart abnormalities such as left ventricular hypertrophy (19.8%), left ventricular dilatation (18.6%), and left ventricular systolic dysfunction (14%) [10].

Anesthetic considerations for the DM patient must involve a thorough assessment of the patient's cardiac status. Patients with cardiac involvement may have an implantable cardioverter defibrillator (ICD) and require interrogation prior to surgery. A defibrillator along with external pads should be available in the perioperative setting.

## 7. Musculoskeletal

DM can affect multiple muscle groups including cardiac, smooth, and skeletal muscle. In a retrospective analysis of 320 patients with DM, Kirzinger et al. found that 14.6% of the patients who underwent general anesthesia had a worsening of musculoskeletal symptoms. This is in part from the effect of hypothermia on exacerbating the myotonia and the prolonged effect of muscle relaxants. Although a majority of these symptoms were reversible, a small group of 9 patients had irreversible aggravation of their disease [6].

## 8. Endocrine

There is an increased incidence in insulin resistance and diabetes particularly in DM2 which should be managed perioperatively with blood glucose measurement as usual for diabetics [1].

## 9. Gastrointestinal

Involvement of the gastrointestinal (GI) system occurs frequently in patients with DM [1, 11]. Dysphagia is prevalent in 25% to 80% of patients with DM. Delayed gastric emptying, choledocholithiasis, irritable bowel syndrome, and elevated gamma glutamyl transferase levels have been associated [11]. The etiology may be from abnormal smooth muscle cells in the alimentary and/or a neurological component. Whether rapid sequence induction and intubation is necessary for all DM patients is unknown.

## 10. Anesthetic Agents and DM

In 2010, Kirzinger et al. published a retrospective study of 134 patients with DM and side effects of anesthesia. 116 of these patients underwent a total of 342 surgical procedures with regional anesthesia over the course of several years [7]. Only 35 of these procedures were performed under spinal or peripheral nerve block. The rest were performed under local anesthesia. From this study, regional anesthetic techniques appear less likely to result in anesthetic complications in the DM patient [7]. This study however did not risk stratify the patients in terms of disease severity. The Myotonic Dystrophy Foundation has provided formalized suggestions for the anesthetic management of patients with Myotonic Dystrophy (see the following). 

*Practical Management*

*Chart Adapted from Myotonic Dystrophy Foundation. Suggestions for Perioperative Management of Patients with DM*

1. Check preoperative blood sugar.
2. Keep patient warm. Use forced-air warming device and increase ambient temperature in OR.
3. Have defibrillator available in the operating room and defibrillator pads on patient.
4. Avoid succinylcholine and neostigmine.
5. Utilize continuous pulse oximetry and EKG monitoring.
6. Plan for possible prolonged postoperative stay.

## 11. Conclusion

DM is a rare genetic disease affecting multiple organs. Here we present a case of a patient with established diagnosis of DM for hip labral arthroscopy. The severity of the patient's disease must be elucidated on preoperative evaluation and is critical for successful anesthetic management. Important considerations and suggestions for management of DM in the preoperative period are presented in this literature review.

## Figures and Tables

**Table 1:** Summary of DM effects on organ systems.

Organ system	Effect	
Musculoskeletal	(i) Myopathy, atrophy, myalgias (ii) Myotonia: triggers include stress and cold as well as specific medications	(i) DM1: tends to affect facial muscles e.g. distal muscles (ii) DM2: tends to affect proximal muscles e.g. hip flexors

Nervous System	(i) Cognitive impairment (ii) Mental retardation more common in DM1	(i) Axonal sensorimotor polyneuropathy (ii) Sensorineural hearing loss

Eye	(i) Cataracts (ii) Proptosis	

Cardiac	(i) Arrhythmias (a) AV block, bundle branch block most common (b) Atrial flutter and fibrillation	(i) Cardiomyopathy: Hypertrophy, dilation, systolic dysfunction

Pulmonary	(i) OSA (ii) Hypersomnia/excessive daytime somnolence (iii) Increased risk of aspiration pneumonia	(i) Respiratory muscle weakness (ii) Increased sensitivity to respiratory depressants

Gastrointestinal	(i) Dysphagia (ii) GERD (iii) IBS-like symptoms	(i) Gallstones

Endocrine	(i) Primary Hypogonadism (ii) Diabetes, Insulin resistance (iii) Hyperthyroidism	(i) Hyperparathyroidism (ii) Hyperhidrosis (iii) Male pattern baldness

Reproductive	(i) Low sperm count secondary to hypogonadism	(i) Higher risk of miscarriage, preterm labor

Cancer	(i) Increased risk for cancers of endometrium, brain, ovary, colon, and skin	

**Table 2:** Summary of DM effects and practice suggestions for perioperative management of patients with DM.

Organ system	Effect	Plan
Central Nervous System	(i) Temperature regulation (ii) Increased risk of corneal abrasions from proptosis	(i) Keep patient warm (ii) Increase temperature in operating room (iii) Careful taping of eyes, ophthalmic ointment

Behavioral issues	(i) Cognitive dysfunction (ii) Behavioral issues (a) Anxiety (b) Bipolar disorder (c) Obsessive- compulsive disorder	(i) Check for mood altering medications (ii) Use small 0.5 mg to 1 mg boluses of benzodiazepines to assess patient's sensitivity

Endocrine	(i) Diabetes (ii) Hyperthyroid (iii) Hyperparathyroid	(i) Check AM blood sugar (ii) Preop thyroid function testing (iii) Check calcium level preoperatively

Cardiac	(i) Arrhythmias: (a) Atrial flutter (b) Atrial fibrillation	(i) Have defibrillator available and pads on patient (ii) Interrogate AICD if present

Pulmonary	(i) Impairment of ventilation and sensitivity to respiratory depressants (ii) Ineffective coughing (a) Aspiration pneumonia	(i) Minimize use respiratory depressants (ii) Aggressive postoperative pulmonary hygiene

Musculoskeletal	(i) Involuntary muscle contraction, progressive weakness	(i) Avoid triggering agents: (a) Succinylcholine (b) Neostigmine

Gastrointestinal	(i) Prolonged gastric emptying	(i) Consider rapid sequence induction and intubation
